# Notes and Notices

**Published:** 1899-07

**Authors:** 


### NOTES AND NOTICES.

Dr. J. B. Gregg Custis announces that on June 29th he will remove his office and residence from 110 East Capitol Street, to 912 Fifteenth Street, McPherson Square, and after that date his office hours will be from 8 until 9 o'clock in the forenoon, and from 3 until 6 o'clock in the afternoon. Washington, D. C. Telephone, 397.

See picture of Alfred Speer on another page, the original wine grower in the United States, whose wines have become famous over the world, also his * * * Climax Grape Brandy.

Dr. Givens' Sanitarium for nervous and mental diseases at Stamford, Conn., offers excellent advantages for patients requiring special care and treatment.

See What a President Says on Brandy for Sickness.—The President of the Baltimore Medical College, who has thoroughly tested Speer's wines and brandy, says: "Speer's Climax Brandy is a pure and valuable article in all cases of disease in which a reliable stimulant is required. I regard it superior to most French brandies."

Dr. B. F. Baily, President of the American Institute of Homœopathy, says: "I have known Dr. Amos J. Givens, of Stamford Hall, Stamford, Conn., for ten years. I have visited his Sanitarium, and have placed patients under his care and treatment, and can recommend Dr. Givens and Stamford Hall in the highest manner."

Also Absolutely Pure Grape Juice.—Speer's Unfermented Grape Juice is perfectly divested of all fermenting principle by electricity and fumigation. His Port, Burgundy, and Claret beat the world for excellence both as a family and medicinal wines.

DR. KIMMELL (who has devoted his entire TIME and PRACTICE to the painless extraction of teeth for the past twenty-one years, using perfectly pure nitrous-oxide manufactured by himself daily) has removed to 1218 Walnut street, and has taken the entire second floor (with all the conveniences) and now has the largest, handsomest, and best furnished offices in Philadelphia. The doctor also administers nitrous-oxide largely for physicians in minor surgical operations. Dr. Kimmel has no students or assistants—all operations in extracting teeth are done personally. Office hours, 8.30 A. M. to 5 P. M.

[The editor of THE HOMŒOPATHIC PHYSICIAN cordially recommends Dr. Kimmell as a skillful and rapid operator, and a careful administrator of nitrous-oxide gas. The doctor has given the most satisfactory service in several cases in the practice of the editor.—W. M. J.]

YELLOW FEVER CONTRACTED AT NIGHT.—A number of convincing instances are related by V. Godinho in a study of yellow fever (*O Brazil Medico*), in which persons escaped yellow fever completely by leaving the infected spots before sundown and sleeping elsewhere at night. At Rio Janeiro and S. Paulo, business can be carried on during the day by non-immunes, without danger of infection, if they have their homes at Petropolis or some other suburb. The head of a prominent German business firm at Santos thus passed unscathed through several epidemics, even the epidemic of 1889, but one night found it more convenient to sleep at Santos and contracted the disease that night. Similar instances are constantly being reported. Sanarelli has demonstrated that the *bacillus icteroides* is destroyed by seven hours' exposure to sunlight. Globig, of the German Navy, recently published a communication (*Arch. de Med. Nav.*) stating that while in port at Rio Janeiro none but the sailors on night duty contracted yellow fever. The popular belief that yellow fever is only contracted after an attack of indigestion Godinho explains by the fact that indigestion and fever are the initial symptoms of the invasion of the infection, caused by the efforts of the system to eliminate toxines.—*Health*, July 15th, 1899.

HAY FEVER.—An Ohio editor says hay fever is caused by kissing grass widows. A Missouri editor says it is caused by a grass widow kissing a fellow by moonlight. An Iowa editor says it is caused by a fellow kissing the hired girl when feeding the cow. An Illinois exchange is of the opinion that it is caused by missing the hired girl and kissing the cow. —*North American Chemical Review.*

All wrong. It is caused by the picnic grass lunch.

A SIMPLE METHOD OF REMOVING A NEEDLE.—"I think it may be of service to record a simple means by which I obtained the removal of a broken needle from the heel of a young lady, aged twelve, whom I saw lately walking about on her toes to avoid her right heel (into which a needle had been broken) touching the ground. The buried end could be felt, but any pressure led to its further entry. I directed her to wear a large, thick corn-plaster around the spot, with a little wet cotton-wool in the centre, and to tread freely on the heel. Within a week afterwards she showed me the needle, which had protruded, and she had easily withdrawn it. Thus no wound was made, and no scar left to be a tender spot on the plantar surface."—*Dr. Charles Steele, in British Medical Journal.*

A CORRESPONDENT of the New York *Sun* wants to know who wrote the following verse:

Beneath a shady tree they sat;
He held her hand, she held his hat;
I held my breath and lay right flat.
They kissed, I saw them do it.
He held that kissing was no crime;
She held her head up every time.
I held my peace and wrote this rhyme:
They thought that no one knew it.

When you think of the inherent meanness of the act of the author, it is no wonder that he, or she, failed to give a name, even as an evidence of good faith.

THE *Public Health Journal* for October has the following clever satire on the "Triumph of Modern Surgery:"

They sawed off his arms and his legs,
They took out his jugular vein;
They put fancy frills on his lungs,
And they deftly extracted his brain.
'Twas a triumph of surgical skill
Such as never was heard of till then;
'Twas the subject of lectures before
Conventions of medical men.
The news of this wonderful thing
Was heralded far and wide;
But as for the patient, there's nothing to say,
Excepting, of course, that he died.

CHEMISTS ORDER SNAKES.—Four hundred live rattlesnakes and copperheads would be a rather startling order, even if received by a zoological garden or the menagerie of the average circus, but consternation reigned among the farmers of Fayette county when H. K. Mulford & Co., of this city, communicated with parties in Uniontown and endeavored to secure the deadly reptiles, which are to be sent here for use in their Philadelphia laboratory, where their virus will be used to inoculate animals and the stricken creatures experimented with in an endeavor to find an antidote for the poison and a cure for the snake-bitten, there now being no known remedy for their bites.

Snakes are plentiful enough in the mountains around Uniontown, but the farmers and mountaineers at first did not greatly relish the idea of entering upon an extended search for that particular form of death. The agent, however, finally succeeded in enlisting the services of a number of the more daring ones, and they will soon fill either that order or the graves of martyrs to the cause of science.—*The Times*, June 15th, 1899.

Electrical Saws.—The Boston Emergency Hospital has adopted the use of an electric circular saw to take the place of the knife in making amputations. Dr. Galvin claims that by its use no anæsthetic is necessary, as the operation is painless, and it shortens the time, as it cuts through almost instantly. This he claims lessens the shock. No ligatures are needed, and all the cases so far treated have done better than by the old method.—*Medical Times.*

---

### FUN FOR DOCTORS.

Temperance Worker.—"My friend, when you have a craving for strong drink you should think of your wife."

Toper.—"Madam, when the desire is upon me I am absolutely devoid of fear."

Expectant Father.—"Well, is it a little peach?"

Excited Nurse.—"No, it's a little pair."—*Ex.*

### Very Profitable.

A Dublin newspaper has an advertisement possibly more truthful than intended: "Wanted, a gentleman to undertake the sale of a patent medicine. The advertiser guarantees that it will be profitable to the undertaker."—*Medical Times.*

### Eliza's Successor.

I'll si'g you a so'g which wo't be lo'g,
All od accou't of Coryza!
I'll tie my charger with a tho'g,
All od accou't of Coryza!
Bud Doble is a doblemad;
He helps a fellow when he cad—
I cad prodouse his nabe, bedad,
All od accou't of Coryza!

"If I get sick, dear, send me to the hospital."

"What! among all those pretty nurses! I guess not!"—*Exchange.*

